# The Impact of Surface CD20 Expression and Soluble CD20 Levels on In Vivo Cell Fragility in Chronic Lymphocytic Leukemia †

**DOI:** 10.3390/jcm14217529

**Published:** 2025-10-24

**Authors:** Ozlem Candan, Imren Tatli, Abdullah Bakisli, Baris Kula, Edanur Korkut, Mehmet Emin Yildirim, Muhammet Ali Gurbuz, Asu Fergun Yilmaz, Isik Atagunduz, Ayse Tulin Tuglular, Tayfur Toptas

**Affiliations:** 1 Division of Hematology, Marmara Faculty of Medicine, Marmara University, 34722 Istanbul, Turkey; imrenaydin@hotmail.com (I.T.); fergunaydin@hotmail.com (A.F.Y.); isikkaygusuz@yahoo.com (I.A.); attuglular@gmail.com (A.T.T.); toptast@gmail.com (T.T.); 2 Marmara Faculty of Medicine, Marmara University, 34722 Istanbul, Turkey; abdullahbakisliresmi@gmail.com (A.B.); kulabaris276@gmail.com (B.K.); edanurkorkut8@gmail.com (E.K.); mehmet.emn.yldrm@gmail.com (M.E.Y.); muhammetaligurbuz99@gmail.com (M.A.G.)

**Keywords:** chronic lymphocytic leukemia, soluble CD20, CD20 expression, smudge cell

## Abstract

**Background:** Patients with chronic lymphocytic leukemia (CLL) who were not receiving treatment were included in this experimental prospective correlation study. We aimed to elucidate the complex relationship between smudge cells, surface CD20, and soluble CD20 in CLL patients. **Methods:** We created blood smears from blood samples collected from our patients using a manual technique consistently performed by the same technician. The May–Grunwald Giemsa dye was used to stain all of the slides. The B-cell phenotypic was analyzed using the FacsCanto II flow cytometer (Becton Dickinson, CA, USA) at the time of diagnosis. Competitive Enzyme-Linked Immunoassay (ELISA) was used to quantitatively assess the amounts of soluble CD20/MS4A1. **Results:** The percentage of smudge cells and soluble CD20 antigen levels were shown to be significantly inversely correlated, suggesting a considerable link (correlation coefficient (r) = −0.51, *p* = 0.006). Similarly, a significant inverse relationship (r = −0.36, *p* = 0.04) was found by the Spearman correlation test between the smudge cell ratio and CD20 median fluorescence intensity (MFI) on cell surfaces. Soluble CD20/MS4A1 and surface CD20 MFI were shown to have a weakly positive association that was almost statistically significant (Spearman’s rho = 0.34, *p* = 0.064). With a sensitivity of 69% and specificity of 86%, we discovered that a cut-off value of 2.2 ng/dL for soluble CD20 predicted higher smudge cells (area under the curve (95% confidence interval (CI)): 0.75 (0.57 to 0.93), *p* = 0.021). **Conclusions:** We found a significant inverse association between smudge cells and both surface CD20 and soluble CD20/MS4A1 in our study examining the correlation between smudge cells, soluble CD20, and CD20/MS4A1 in CLL patients. Our findings indicate that soluble CD20 may contribute to understanding the pathophysiology of smudge cells and could be further investigated as a potential prognostic marker in CLL.

## 1. Introduction

Chronic lymphocytic leukemia (CLL) accounts for approximately 25–30% of all leukemias in Western countries, with an incidence rate of about 4–6 cases per 100,000 persons annually [1]. The condition primarily affects older individuals [2]. With improvements in disease management, the 5-year relative survival rate for CLL patients improved from 65.1% in 1975 to a projected 87.2% in 2021 [3]. CLL treatment has shifted from chemoimmunotherapy (CIT) to targeted novel agents such as Bruton Tyrosine Kinase inhibitors (BTKi; ibrutinib, acalabrutinib, zanubrutinib) and the B-cell lymphoma 2 (BCL2) inhibitor venetoclax. These targeted therapies are now approved as monotherapies or combined with anti-CD20 monoclonal antibodies. Their synergistic effects have naturally prompted the investigation of novel combination therapies involving multiple new agents [4,5,6].

Smudge cells (SCs), also called Gumprecht shadows, smear cells, or basket cells, are fragmented CLL B cells found in the blood smears of CLL patients. They do not possess any discernible cytoplasmic membrane or nuclear structure. Studies by Nowakowski et al. have demonstrated that the proportion of SCs in routine blood smears is an independent prognostic indicator in CLL patients [7,8]. Another study found that 66.7% of newly diagnosed Egyptian B-CLL patients had a smudge cell percentage below 30%, with a significant correlation between a low smudge cell percentage (below 30%) and advanced-stage disease. It was also determined that patients with more than 30% smudge cells at diagnosis had higher survival rates [9]. Similarly, research by Sall A et al. showed that, in line with the suggestion by Nowakowski et al. [8], using a 30% cut-off value for SC percentage is associated with disease prognosis [10]. The mechanism underlying the formation of SCs remains unclear. Nowakowski et al. have identified an inverse relationship between the formation of SCs and vimentin, a cytoskeletal protein [7]. They have explained this finding by noting that high vimentin expression in lymphocytes is associated with increased cellular rigidity [11].

In patients with CLL, it is thought that soluble CD20 may originate from the breakdown of leukemic cells. It has been previously hypothesized that soluble CD20 might play a role in SC formation [8]. In this analysis, we explored the possible relationship between smudge cells, surface CD20, and soluble CD20 in patients with CLL.

## 2. Materials and Methods

### 2.1. Patients

This study included 30 treatment-naïve CLL patients who were diagnosed and followed at the Hematology Department of Marmara University Hospital between December 2022 and June 2023. Eligibility criteria included diagnosis confirmed by flow cytometry, absence of prior anti-leukemic therapy, and no concurrent malignancy or active infection. The sample size (*n* = 30) was determined considering the accessibility of eligible patients and the minimum statistical threshold required for correlation studies.

The diagnosis of CLL was made by flow cytometry, which confirmed the presence of at least 5000/μL circulating monoclonal B cells with a CLL immunophenotype which includes the expression of CD19, CD5, and CD23; weak expression of CD20; and either kappa or lambda light chains. In addition, there may be expression of CD43 and CD200, but CD79b is expected to be weak or missing [12].

Every participant gave informed consent, and the research followed the Helsinki Declaration’s strict guidelines for clinical practice. The Institutional Ethics Committee approved the study (Approval number: 09.2022.1637). We recorded demographic characteristics; laboratory data including leukocyte, lymphocyte, hemoglobin, and platelet counts; and genetic test results from the hospital’s computerized database.

### 2.2. Peripheral Blood Smears of the Patients

We prepared blood smears from blood samples using a manual technique consistently performed by the same technician. The May–Grunwald Giemsa dye was used to stain the slides. The sample from each patient was split into two slides, which were inspected by the same technician who did not know about the patient’s progress or the clinical facts. The findings from the two assessments were compared in order; if there was a significant difference between the assessments that went beyond three percent, a third assessment was conducted, and the average of the three assessments was calculated.

The proportion of SCs was assessed by calculating the proportion of total lymphocytes, rather than as a percentage of total white blood cells (WBC), using the following formula: Number of SCs × 100/Total lymphocytes (SCs + intact lymphocytes).

### 2.3. Immunophenotyping

The B-cell phenotypic was analyzed using the FacsCanto II flow cytometer (Becton Dickinson, CA, USA) at the time of diagnosis. Using this method, the levels of CD20 MFI in CLL cells were determined.

### 2.4. Soluble CD20/MS4A1 (Membrane Spanning 4-Domains A1) Level Analysis via Competitive ELISA (Enzyme-Linked Immunoassay)

A 5cc blood sample was withdrawn from all patients and healthy subjects. It was then centrifuged at room temperature (3000 rpm), and the resulting serum samples were stored at −80 °C until subsequent analysis. The competitive ELISA kit, cataloged as LG-EH14877 by FARMASINA, was utilized. This method employs a competitive ELISA protocol to quantitatively determine levels of soluble CD20/MS4A1. Dispensing specific antibodies against soluble CD20/MS4A1 into 96-well plates allowed free soluble CD20 and biotin-bound CD20 to compete for binding. The plates are then filled with the streptavidin–Horseradish Peroxidase (HRP) combination after washing procedures to eliminate unbound antibodies. Tetramethylbenzidine, a particular substrate, is then introduced, starting a colorimetric reaction that is catalyzed by the HRP enzyme. The reaction is stopped with the addition of a stop solution, and the amount of soluble CD20/MS4A1 is quantified by measuring absorbance at 450 nm. The recognized unit indicating the soluble CD20/MS4A1 levels was ng/dL.

### 2.5. Data Analysis

The statistical analysis was conducted using Statistical Package for the Social Sciences (SPSS) 28.0.1.1 software. Frequency and cross-tabulation tables were investigated in these investigations. The Shapiro–Wilk Test was used to determine whether the data distribution was normal. For skewed data, non-parametric tests including the Spearman correlation and the Kruskal–Wallis test were used. A significance level of *p* < 0.05 was adopted. The Receiver Operating Characteristic (ROC) curve was employed to establish the threshold value for soluble CD20, which predicts the occurrence of smudge formation in CLL patients.

## 3. Results

Patients ranged in age from 47 to 83 years, with 20 out of 30 (66.7%) being male. The median age of the patient population was 65 years. Thirty of the study participants had at least three of the B symptoms (fever, sweats at night, and weight loss) at the time of diagnosis. Lymphadenopathy was observed in 11 out of 30 (36%) of the patients, while six out of 30 (20%) had hepatosplenomegaly. The patients were divided into the following Rai stages: 13 patients (43.3%) were in stage 0, 7 patients (23.3%) were in stage 1, 3 patients (10%) were in stage 2, 6 patients (20%) were in stage 3, and 1 patient (3.3%) was in stage 4 (Table 1).

Median white blood cell, lymphocyte count, hemoglobin value, and platelet count were 27.5 × 10^3^/µL, 21.9 × 10^3^ cells/µL, 13.05 g/dL, and 184 × 10^3^/µL (Table 1). In flow cytometric analysis, median CD20 MFI on cell surfaces was 227. Soluble CD20/MS4A1 levels in competitive ELISA analysis were 2.11 ng/dL. Median percentage of SCs on each high-power field was 25.75% (Table 2).

**Figure 1:**
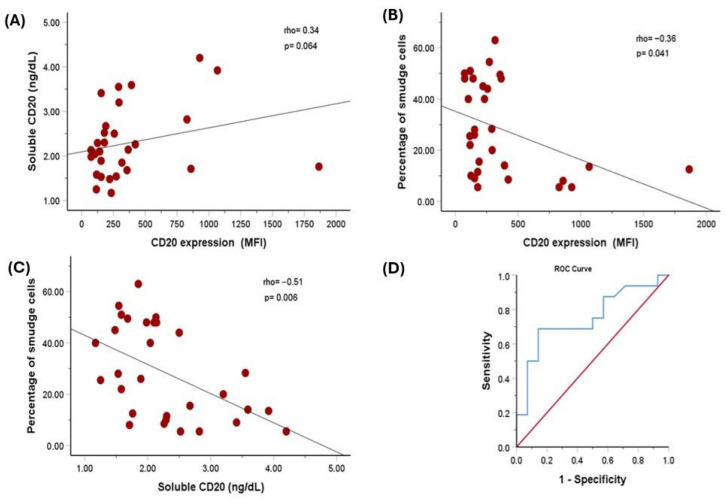
(**A**) A weak positive relationship was found between soluble CD20/MS4A1 and surface CD20 MFI. (**B**) Spearman correlation test demonstrated a significant inverse association between CD20 MFI on cell surfaces and the smudge cell ratio. (**C**) There was a significant inverse relationship between the levels of soluble CD20 antigen and the percentage of smudge cells, indicating a moderate relationship. (**D**) These findings show that the cut-off value of soluble CD20 predicting smudge cell formation is 2.2 ng/dL, and this value is statistically significant.

A weak positive relationship was found between soluble CD20/MS4A1 and surface CD20 MFI, but it was on the edge of statistical significance (Spearman’s rho = 0.34, *p* = 0.064). Spearman’s correlation test revealed a significant inverse association between CD20 MFI on cell surfaces and the smudge cell ratio (r = −0.36, *p* = 0.04). Additionally, a significant inverse relationship was observed between levels of soluble CD20 antigen and the percentage of smudge cells, indicating a moderate correlation (correlation coefficient (r) = −0.51, *p* = 0.006) (Figure 1A–C and Table 3). To determine the cut-off value for soluble CD20 predicting smudge formation in CLL patients, we performed ROC curve analysis. The median percentage of smudge cells, which was 25.75%, was established as the cut-off value. With a sensitivity of 69% and specificity of 86% (area under the curve (95% CI): 0.75 [0.57 to 0.93], *p* = 0.021), soluble CD20 at a cut-off of 2.2 ng/dL predicted more smudge cells (Figure 1D).

## 4. Discussion

In this investigation, the percentage of SCs and soluble CD20 antigen levels were found to be significantly inversely correlated. Similarly, we found an inverse relationship between the amount of surface CD20 antigen and the smudge cell ratio. Further, we demonstrated that a cut-off value for soluble CD20 of 2.2 ng/dL or less predicts smudge cell formation. A weak positive relationship was found between soluble CD20/MS4A1 and surface CD20 MFI. These observations may help explain the mechanisms underlying smudge cell formation.

Smudge cells, occasionally detected in normal peripheral blood films (PBFs) [13], are also commonly present in abnormal PBFs, particularly among individuals diagnosed with acute and chronic leukemia, though typically in higher concentrations in CLL [14]. The exact process responsible for the presence of SCs in peripheral blood films is not well known.

According to Nowakowski et al., soluble CD20 in CLL patients’ plasma may be liberated from leukemic cells, and membrane shedding may result in the development of smudge cells. They also hypothesized that elevated levels of soluble CD20 might be associated with a poor prognosis in CLL [8]. However, they did not test this hypothesis. Our findings demonstrated that soluble CD20 in the plasma of CLL patients is inversely related with smudge cell formation. Although the exact mechanism of CD20 antigens is unknown, we postulate that most probably CD20 antigens reduce the fragility of leukemic cells by supporting the cytoskeletal proteins. Our results support the hypothesis that surface CD20 expression and soluble CD20 levels are interrelated. Both of those parameters predict in vivo cell fragility.

A previous study pointed out that CD20 expression is related with trisomy 12 and good response to rituximab therapy [15]. A separate investigation examining the influence of CD20 expression on disease outcomes found that individuals with newly diagnosed, untreated chronic lymphocytic leukemia (CLL) with high levels of CD20 expression experience improved survival without the need for therapy. However, they could not demonstrate any relationship between CD20 expression and rituximab response [8]. So, the conclusions of studies assessing the impact of CD20 expression on survival are conflicting. Several confounders should have been considered. Since we did not perform survival analyses, we cannot draw a definite conclusion regarding the impact of CD20 expression or soluble CD20 levels on disease outcomes. However, our findings support the hypothesis that soluble CD20 may influence disease progression and treatment response in CLL. Previous studies have shown that soluble CD20 can originate from membrane shedding of leukemic cells, potentially acting as a decoy antigen that interferes with anti-CD20 antibody binding [16]. Increased soluble CD20 levels may therefore reflect enhanced leukemic cell turnover or membrane instability. Consistent with our results, prior work has also demonstrated that cellular fragility markers such as smudge cells correlate with disease outcomes in CLL [8], further supporting the potential of soluble CD20 as a prognostic biomarker.

The mechanism of smudge cell formation has a key role in the pathophysiology of lymphocytic leukemia. It is believed to be associated with changes in the structural properties of lymphocytes that make them more fragile or susceptible to fragmentation than normal [14,17,18]. In this study, we provided a cut-off value for soluble CD20, which could potentially aid in predicting the extent of smear cell presence.

The small sample size is a limitation of this study. However, the primary aim was to examine the relationships between laboratory parameters. Nevertheless, it was sufficient to demonstrate significant relationships. Due to the prolonged overall survival of CLL patients, there was insufficient observation time to evaluate the effects of smudge cell count, surface CD20, and soluble CD20 levels on prognosis and overall survival.

## 5. Conclusions

There is a significant and inverse relationship between smudge cells and both surface CD20 and soluble CD20/MS4A1. Soluble CD20 and surface CD20 expression are correlated. Both of those markers may be related to in vivo cell fragility.

## Figures and Tables

**Table 1:** Patient Characteristics (N = 30).

Characteristic	Value
Age, median (range), years	65 (47–83)
Male, *n* (%)	20 (66.7)
WBC (×10^3^/μL), median (range)	27.5 (8.3–209.9)
Lymphocyte (×10^3^/μL), median (range)	21.9 (5.4–191.8)
Hemoglobin (g/dL), median (range)	13.05 (9.2–16)
Platelet (×10^3^/μL), median (range)	184 (107–358)
RAI, *n* (%)	
0	13 (43.3)
I	7 (23.3)
II	3 (10.0)
III	6 (20.0)
IV	1 (3.3)
IgHV, *n* (%)	
Mutated	13 (43.3)
Unmutated	11 (36.6)
Unknown	6 (20)

WBC: White Blood Cell; per μL: per microliter; IgHV: immunoglobulin heavy chain.

**Table 2:** Patient Characteristics.

**Variable**	**Value**
CD20 MFI, median (range)	227 (74–1864)
Soluble CD20/MS4A1(ng/dL), median (range)	2.11 (1.17–4.20)
Percentage of smudge cells, median (range)	25.75 (5.5–63)

MFI, Median fluorescence intensity; MS4A1, Membrane Spanning 4-Domains A1.

**Table 3:** The relationship between the percentage of smudge cells and other laboratory tests.

Variable 1	Variable 2	Correlation Coef (rho)	*p* Value
Soluble CD20	Percentage of smudge cells	−0.51	0.006
CD20 MFI	Percentage of smudge cells	−0.36	0.041
Soluble CD20	CD20 MFI	0.34	0.064

MFI, Median fluorescence intensity; Correlation coef. (rho), correlation coefficient (Spearman’s rho).

## Data Availability

The raw data supporting the conclusions of this article will be made available by the authors on request.
